# Supplementary material for: Sustainable Extraction of Prospective Cosmetic Ingredients from Colombian Marine Macroalgae Using Natural Deep Eutectic Solvents
Source: Mar Drugs. 2025 May 30;23(6):239. doi: 10.3390/md23060239 (PMC12194361; doi:10.3390/md23060239)
Supplement: Supplementary file 1 [file marinedrugs-23-00239-s001.zip › marinedrugs-3566558-supplementary.pdf]

Supporting information

Sustainable extraction of prospective cosmetic ingredients from Colombian marine macroalgae using natural deep eutectic solvents

Macroalgae species	Mass of raw material [mg]	Volume of extract [mL]	Concentration of extract in NADES [ug/mL]	Slope (mL/ug)	Y-Intercept	Mean Abs	mg GAE/g DW
Ceramium nitens	104,7	1,5	309,6	0,0008	-0,0097	0,238	4,44
	104,1	1,5	270,9	0,0008	-0,0097	0,207	3,90
	103,1	1,5	232,1	0,0008	-0,0097	0,176	3,38
Dictyota menstrualis	103,5	1,5	854,6	0,0008	-0,0097	0,674	12,4
	101,3	1,5	823,4	0,0008	-0,0097	0,649	12,2
	103,5	1,5	825,9	0,0008	-0,0097	0,651	12,0
Dictyota cf. *pulchella* a	101,6	1,5	575,9	0,0008	-0,0097	0,451	8,50
	101,4	1,5	550,9	0,0008	-0,0097	0,431	8,15
	102,8	1,5	548,4	0,0008	-0,0097	0,429	8,00
Dictyota cf. *pulchella* b	102,7	1,5	497,1	0,0008	-0,0097	0,388	7,26
	101,8	1,5	475,9	0,0008	-0,0097	0,371	7,01
	100,4	1,5	502,5	0,0008	-0,0097	0,392	7,51
Gracilariopsis lemaneiformis	104,0	1,5	154,6	0,0008	-0,0097	0,114	2,23
	101,7	1,5	153,8	0,0008	-0,0097	0,113	2,27
	102,8	1,5	152,1	0,0008	-0,0097	0,112	2,22
Hypnea sp. 1	101,2	1,5	343,4	0,0008	-0,0097	0,265	5,09
	104,1	1,5	335,0	0,0008	-0,0097	0,258	4,83
	102,7	1,5	364,6	0,0008	-0,0097	0,282	5,33
Hypnea sp. 2	100,8	1,5	264,2	0,0008	-0,0097	0,202	3,93
	101,0	1,5	306,3	0,0008	-0,0097	0,235	4,55
	101,3	1,5	287,1	0,0008	-0,0097	0,220	4,25
Pterocladiella capillacea	102,6	1,5	378,8	0,0008	-0,0097	0,293	5,54
	103,8	1,5	309,2	0,0008	-0,0097	0,238	4,47
	103,3	1,5	456,7	0,0008	-0,0097	0,356	6,63
Sargassum ramifolium	102,8	1,5	1185	0,0008	-0,0097	0,938	17,3
	104,4	1,5	1206	0,0008	-0,0097	0,955	17,3

	101,5	1,5	1165	0,0008	-0,0097	0,922	17,2
Sargassum fluitans	102,8	1,5	1039	0,0008	-0,0097	0,821	15,2
	102,0	1,5	1026	0,0008	-0,0097	0,811	15,1
	102,4	1,5	1033	0,0008	-0,0097	0,817	15,1
Solieria filiformis	103,8	1,5	342,1	0,0008	-0,0097	0,264	4,94
	103,7	1,5	342,1	0,0008	-0,0097	0,264	4,95
	102,5	1,5	337,1	0,0008	-0,0097	0,260	4,93
Stypopodium zonale	100,9	1,5	244,6	0,0008	-0,0097	0,186	3,64
	104,4	1,5	205,5	0,0008	-0,0097	0,155	2,95
	101,8	1,5	291,7	0,0008	-0,0097	0,224	4,30

Table *S1 Results of determination of the content of phenolic compounds in extracts from algae using the NADES FGlcW115 (r2 = 0.958)*

**Macroalgae species**	**Mass of raw material [mg]**	**Volume of extract [mL]**	**Concentration of extract in NADES [ug/mL]**	**Slope (mL/ug)**	**Y-Intercept**	**Mean Abs**	**mg GAE/g DW**
Ceramium nitens	102,2	1,5	904,0	0,0004	0,0024	0,364	13,3
	102,9	1,5	891,5	0,0004	0,0024	0,359	13,0
	101,9	1,5	918,2	0,0004	0,0024	0,370	13,5
Dictyota menstrualis	101,5	1,5	1257	0,0004	0,0024	0,505	18,6
	101,2	1,5	1295	0,0004	0,0024	0,520	19,2
	103,2	1,5	1236	0,0004	0,0024	0,497	18,0
Dictyota cf. *pulchella* a	101,4	1,5	1343	0,0004	0,0024	0,540	19,9
	104,1	1,5	1516	0,0004	0,0024	0,609	21,8
	102,6	1,5	1427	0,0004	0,0024	0,573	20,9
Dictyota cf. *pulchella* b	101,0	1,5	788,2	0,0004	0,0024	0,318	11,7
	104,1	1,5	859,8	0,0004	0,0024	0,346	12,4
	102,9	1,5	826,5	0,0004	0,0024	0,333	12,0
Gracilariopsis lemaneiformis	102,0	1,5	174,0	0,0004	0,0024	0,072	2,56
	103,0	1,5	146,5	0,0004	0,0024	0,061	2,13
	104,0	1,5	151,5	0,0004	0,0024	0,063	2,19

Hypnea sp. 1	102,0	1,5	735,7	0,0004	0,0024	0,297	10,8
	100,0	1,5	721,5	0,0004	0,0024	0,291	10,8
	104,8	1,5	754,8	0,0004	0,0024	0,304	10,8
Hypnea sp. 2	103,4	1,5	308,0	0,0004	0,0024	0,126	4,47
	102,8	1,5	324,8	0,0004	0,0024	0,132	4,74
	102,5	1,5	287,3	0,0004	0,0024	0,117	4,20
Pterocladiella capillacea	104,0	1,5	842,3	0,0004	0,0024	0,339	12,1
	100,0	1,5	782,3	0,0004	0,0024	0,315	11,7
	103,0	1,5	819,0	0,0004	0,0024	0,330	11,9
Sargassum ramifolium	101,0	1,5	1965	0,0004	0,0024	0,789	29,2
	103,0	1,5	1890	0,0004	0,0024	0,758	27,5
	100,0	1,5	2057	0,0004	0,0024	0,825	30,9
Sargassum fluitans	102,0	1,5	1471	0,0004	0,0024	0,591	21,6
	102,0	1,5	1512	0,0004	0,0024	0,607	22,2
	100,0	1,5	1457	0,0004	0,0024	0,585	21,8
Solieria filiformis	102,0	1,5	520,3	0,0004	0,0024	0,211	7,65
	102,0	1,5	506,5	0,0004	0,0024	0,205	7,45
	102,0	1,5	535,7	0,0004	0,0024	0,217	7,88
Stypopodium zonale	101,0	1,5	574,8	0,0004	0,0024	0,232	8,54
	102,0	1,5	631,5	0,0004	0,0024	0,255	9,29
	103,0	1,5	686,5	0,0004	0,0024	0,277	10,0

Table *S2 Results of determination of the content of phenolic compounds in extracts from algae using the NADES BGlcW115 (r2 = 0.9992)*

**Macroalgae species**	**Mass of raw material [mg]**	**Volume of extract [mL]**	**Concentration of extract in NADES [ug/mL]**	**Slope (mL/ug)**	**Y-Intercept**	**Mean Abs**	**mg GAE/g DW**
Ceramium nitens	101,8	1,5	509,3	0,0004	0,0013	0,205	7,50
	100,4	1,5	526,8	0,0004	0,0013	0,212	7,87
	100,1	1,5	514,3	0,0004	0,0013	0,207	7,71
Dictyota menstrualis	104,1	1,5	1383	0,0004	0,0013	0,555	19,9
	102,2	1,5	1382	0,0004	0,0013	0,554	20,3

	102,9	1,5	1418	0,0004	0,0013	0,568	20,7
Dictyota cf. *Pulchella* a	102,8	1,5	996,8	0,0004	0,0013	0,400	14,5
	103,6	1,5	1139	0,0004	0,0013	0,457	16,5
	101,1	1,5	1047	0,0004	0,0013	0,420	15,5
Dictyota cf. *pulchella* b	101,3	1,5	605,9	0,0004	0,0013	0,244	8,97
	102,7	1,5	851,8	0,0004	0,0013	0,342	12,4
	101,6	1,5	726,8	0,0004	0,0013	0,292	10,7
Gracilariopsis lemaneiformis	101,0	1,5	208,4	0,0004	0,0013	0,085	3,10
	100,0	1,5	166,8	0,0004	0,0013	0,068	2,50
	105,0	1,5	130,1	0,0004	0,0013	0,053	1,86
Hypnea sp. 1	103,1	1,5	419,3	0,0004	0,0013	0,169	6,10
	103,9	1,5	446,8	0,0004	0,0013	0,180	6,45
	100,1	1,5	454,3	0,0004	0,0013	0,183	6,81
Hypnea sp. 2	101,1	1,5	301,8	0,0004	0,0013	0,122	4,48
	101,2	1,5	294,3	0,0004	0,0013	0,119	4,36
	102,1	1,5	287,6	0,0004	0,0013	0,116	4,23
Pterocladiella capillacea	104,0	1,5	793,4	0,0004	0,0013	0,319	11,4
	101,0	1,5	922,6	0,0004	0,0013	0,370	13,7
	105,0	1,5	879,3	0,0004	0,0013	0,353	12,6
Sargassum ramifolium	101,0	1,5	527,6	0,0004	0,0013	0,212	7,84
	105,0	1,5	411,8	0,0004	0,0013	0,166	5,88
	103,0	1,5	471,8	0,0004	0,0013	0,190	6,87
Sargassum fluitans	103,0	1,5	1127	0,0004	0,0013	0,452	16,4
	101,0	1,5	906,8	0,0004	0,0013	0,364	13,5
	102,0	1,5	1079	0,0004	0,0013	0,433	15,9
Solieria filiformis	103,0	1,5	339,3	0,0004	0,0013	0,137	4,94
	101,0	1,5	300,9	0,0004	0,0013	0,122	4,47
	103,0	1,5	324,3	0,0004	0,0013	0,131	4,72
Stypopodium zonale	106,0	1,5	429,3	0,0004	0,0013	0,173	6,07
	103,0	1,5	475,9	0,0004	0,0013	0,192	6,93
	102,0	1,5	441,8	0,0004	0,0013	0,178	6,50

Table *S3 Results of determination of the content of phenolic compounds in extracts from algae using the NADES UGly13 (r2 = 0.9942)*
